# Morpholino artifacts provide pitfalls and reveal a novel role for pro-apoptotic genes in hindbrain boundary development

**DOI:** 10.1016/j.ydbio.2010.11.030

**Published:** 2011-02-15

**Authors:** Sebastian S. Gerety, David G. Wilkinson

**Affiliations:** Division of Developmental Neurobiology, MRC National Institute for Medical Research, The Ridgeway, Mill Hill, London NW7 1AA, UK

**Keywords:** Morpholino, Rhombomere, Toxicity, Neurogenesis, Boundary, Zebrafish

## Abstract

Morpholino antisense oligonucleotides (MOs) are widely used as a tool to achieve loss of gene function, but many have off-target effects mediated by activation of Tp53 and associated apoptosis. Here, we re-examine our previous MO-based loss-of-function studies that had suggested that Wnt1 expressed at hindbrain boundaries in zebrafish promotes neurogenesis and inhibits boundary marker gene expression in the adjacent para-boundary regions. We find that Tp53 is highly activated and apoptosis is frequently induced by the MOs used in these studies. Co-knockdown of Tp53 rescues the decrease in proneural and neuronal marker expression, which is thus an off-target effect of MOs. While loss of gene expression can be attributed to cell loss through apoptotic cell death, surprisingly we find that the ectopic expression of hindbrain boundary markers is also dependent on Tp53 activity and its downstream apoptotic effectors. We examine whether this non-specific activation of hindbrain boundary gene expression provides insight into the endogenous mechanisms underlying boundary cell specification. We find that the pro-apoptotic Bcl genes puma and bax-a are required for hindbrain boundary marker expression, and that gain of function of the Bcl-caspase pathway leads to ectopic boundary marker expression. These data reveal a non-apoptotic role for pro-apoptotic genes in the regulation of gene expression at hindbrain boundaries. In light of these findings, we discuss the precautions needed in performing morpholino knockdowns and in interpreting the data derived from their use.

## Research Highlights

► Previous studies have shown that antisense morpholino oligonucleotides (MOs) can have off-target effects mediated by activation of Tp53 and associated apoptosis. ► We show that in the zebrafish hindbrain, MOs can also non-specifically induce ectopic boundary marker gene expression. ► The induction of ectopic gene expression by MOs is due to a normal non-apoptotic role of the Bcl–caspase pathway that is required for hindbrain boundary gene expression. ► These findings reveal that a previous study suggesting that Wnt1 promotes neurogenesis and restricts hindbrain boundary marker expression is incorrect. ► The finding that MOs can induce non-apoptotic roles of the pro-apoptotic pathway further emphasizes the need for precautions in their use and interpretation of results.

## Introduction

Critical to the application of reverse genetics in developmental biology is the availability of tools to modify gene expression. The technical difficulty of performing a reverse genetic manipulation needs to be weighed against the robustness and reliability of achieving an interpretable outcome. Gene knock-outs and knock-ins in the mouse, for example, require lengthy procedures from construct assembly to blastocyst injection and chimera screening. The advantages, including the generation of true null alleles, faithful reporter gene expression, and more advanced gene modification (for example, conditional alleles and gene replacements), justify this effort.

While the zebrafish *Danio rerio* is well established as a model organism, its appeal as a system for reverse genetic studies was greatly increased by the development of antisense interference using morpholino oligonucleotides (MOs, Nasevicius and Ekker, 2000). These short antisense oligonucleotide analogs are typically designed to bind at or near the translational initiation site of an mRNA of interest, thereby sterically blocking translation. They have also been shown to disrupt mRNA maturation when targeted at pre-mRNA splice sites (Draper et al., 2001; Morcos, 2007), thereby blocking proper expression. Where antibodies were available, it has been repeatedly shown that morpholinos can effectively reduce (“knockdown”) protein levels, and thereby recapitulate the phenotypes of known genetically mutant zebrafish lines. One can not only eliminate gene products selectively but generate hypomorphic embryos as well, by varying the morpholino dose. This, their ease of use and apparent reliability, provides an attractive and widely used approach to reverse genetic studies in a vertebrate model.

Since the earliest publications describing the use of morpholinos in zebrafish, however, studies from Ekker and colleagues have found that 15–20% of morpholinos can exhibit non-specific toxicity in the developing embryo (Ekker and Larson, 2001; Heasman, 2002; Nasevicius and Ekker, 2000). Their recent work (Robu et al., 2007) has further highlighted the drawbacks of using morpholinos and identified apoptosis as a key component of their off-target effects. The tumor suppressor Tp53 is a tightly regulated transcriptional regulator with important roles in the maintenance of genome integrity, DNA repair, and apoptosis of damaged and abnormal cells. Activation of Tp53 by radiation, chemical toxicity, or trophic factor withdrawal can cause cell cycle arrest and apoptotic cell death (Cheng et al., 1997; Vogelstein et al., 2000; Vousden and Prives, 2009). Robu et al. showed that Tp53 activation was responsible for the extensive non-specific cell death seen in many morpholino-injected (morphant) embryos. By knocking down Tp53, they were able to rescue morphant phenotypes that did not match those of the corresponding mutant fish lines. In these examples, loss of particular tissues was observed, and the non-specific phenotypes were the result of apoptotic cell death.

In any manipulation where the resulting phenotype is loss of a cell type, domain or tissue, apoptosis is often a contributing factor, and consequently cell death is routinely assessed. Numerous mutant mouse lines display developmental defects that are partially or completely dependent on apoptosis, and specific aspects of the phenotype are rescued by crossing into a Tp53 null background (Hettmann et al., 2000; Jones et al., 1995; Montes de Oca Luna et al., 1995; Morgenbesser et al., 1994; Sugo et al., 2004). By performing an analogous experiment in zebrafish, phenotypic analysis can be done in the absence of Tp53, thereby assessing the contribution of apoptosis to the observed phenotypes (Chen et al., 2005; Langheinrich et al., 2002; Robu et al., 2007). Because morpholinos can cause non-specific apoptosis, however, a Tp53 knockdown cannot distinguish between target dependent cell death and a non-specific effect of morpholino toxicity. Verifying the specificity of a cell-death phenotype identified by morpholino knockdown requires an alternative method, such as a germline mutant, deletion, or other such loss of function. The use of morpholinos therefore has a “blind spot”, where a category of phenotypes is not interpretable without independent confirmation.

In this study, we revisit previous work from our laboratory (Amoyel et al., 2005), taking into account recent advances in understanding of morpholino toxicity. This previous study analysed the function of Wnt1, which is transiently expressed at rhombomere boundaries, in relation to neurogenesis, which occurs in non-boundary regions of the hindbrain. It was found that MO-mediated knockdown of Wnt1 led to decreased neurogenesis, and this effect was reversed by overexpression of dominant active beta-catenin. Strikingly, Wnt1 knockdown led to ectopic expression of hindbrain boundary markers in a temporally and spatially restricted pattern: boundary marker expression was initially restricted as normal, but after 18 h of development spread throughout the hindbrain, except in rhombomere 4 (r4). Based on finding similar phenotypes following knockdown of proneural and delta genes, a network of interactions was proposed in which Wnt1 serves to promote proneural gene expression, which in turn prevents the spreading of boundary cell identity into non-boundary regions.

Here, we report that the decrease in proneural and neuronal marker expression is due to off-target effects of MOs that activate the Tp53-mediated cell death pathway. Surprisingly, we find that many MOs also non-specifically induce ectopic expression of boundary markers. In rescue and drug-induced apoptosis experiments, we show that the reciprocal changes to neuronal and boundary marker expression are not because these genes regulate each other, but rather are each due to activation of Tp53 and apoptosis effectors. We have analysed whether the non-specific induction of boundary marker expression in morpholino injected embryos reflects an endogenous mechanism of boundary cell regulation. We provide evidence that specific pro-apoptotic genes are required for expression of markers of hindbrain boundaries, indicative of a non-apoptotic role. These data provide an explanation for why morpholino toxicity leads to ectopic gene expression: Tp53 activation throughout the hindbrain results in widespread activation of pro-apoptotic genes, thus driving the normal program of boundary gene expression ectopically. These data provide the first description of a non-apoptotic role for pro-apoptotic genes in boundary formation. Our finding that off-target effects of MOs can induce specific gene expression raises issues of wider concern, since MOs are being used by many laboratories to study gene function and regulatory networks. We discuss strategies to ensure that results obtained when using MOs are not due to non-specific effects.

## Materials and methods

### Fish maintenance

Wild-type and transgenic zebrafish embryos were obtained by natural spawning and raised at 28.5 °C, as described (Westerfield, 1993). All injections were performed with control MO and/or uninjected controls where indicated and were collected from the same laying as experimental injections.

### Transgenic and mutant zebrafish lines

The Krox20::GAL4line was a generous gift of Reinhard Koster (Distel et al., 2009) and expresses GAL4 specifically in rhombomeres 3 and 5 (r3 and r5). The UAS::puma line was created by inserting the puma CDS (Kratz et al., 2006) into a custom miniTol2 (Balciunas et al., 2006) site-flanked, dual UAS vector, expressing H2B-citrine from one 5xUAS sequence (Nikolaou et al., 2009), and puma from the second 5xUAS. This construct also contains the zebrafish alpha crystallin promoter driving RFP as a genotyping aid to identify carriers. The plasmid was injected with Tol2 transposase mRNA (Balciunas et al., 2006), and carriers were isolated by RFP fluorescence within the retina at 4 days post-fertilization. An auto-activated zebrafish Caspase3a (Rev-Casp3a) was constructed based on the published human version (Srinivasula et al., 1998). This coding sequence was placed under control of a 5xUAS sequence exactly as described for puma above. Krox20::GAL4;UAS::puma,UAS::H2B-Citrine and Krox20::GAL4;UAS::Rev-Casp3a,UAS::H2B-Citrine double transgenic embryos were identified by the green fluorescence of GAL4 driven H2B-Citrine in r3 and r5. The tp53 mutant line (Berghmans et al., 2005) was acquired from the Zebrafish International Resource Center (University of Oregon, Eugene, USA).

### Morpholino oligonucleotides and mRNA injections

Morpholino oligonucleotides (MO) were purchased from Gene Tools (Oregon, USA). All morpholinos were dissolved in water for 5 min at 65 °C, at 1 mM and aliquoted before storing frozen. Before use, morpholinos were thawed for 5 min at 65 °C, cooled on ice, then centrifuged. One- to four-cell embryos were microinjected with 1.8 nl of morpholino diluted in water. All experiments were quantified over at least two independent replicates. The sequences of some morpholinos were described previously and used at the indicated doses: wnt1 (0.51 pmol), ascl1a (0.45 pmol), ascl1b (0.45 pmol), neurog1 (0.45 pmol), deltaA (0.45 pmol, Amoyel et al., 2005) tcf3b (0.36 pmol, Dorsky et al., 2003), rfng (0.56 pmol, TGGAGGCGACATGGGATAAGTGCAT, Cheng et al., 2004), tp53 (0.45–0.79 pmol GCGCCATTGCTTTGCAAGAATTG, Langheinrich et al., 2002), puma splice blocker (puma SB MO, 0.56 pmol, Sidi et al., 2008), puma ATG blocker (puma ATG MO, 0.56 pmol, Kratz et al., 2006), bax-a (0.54 pmol, Kratz et al., 2006).

The efficacy of the splice-blocking puma MO was tested in two ways: first, by RT-PCR we confirmed that puma transcript splicing was blocked by puma SB MO injection but not control MO injection (Supplemental Fig. 3 A,B. Primer sequences are F:ATG GCC CGA CCA GAG ATG GAA AG, R1:CTG TGA ATG TAA CCA AGC ATG ACT C, R2:TCA TCT GAG GCC GTG CTG GTA G). Second, we confirmed that Puma depletion by MO injection abrogates gamma irradiation-induced apoptosis in zebrafish embryos (12.5 gray, Sidi et al., 2008, and Supplemental Fig. 3C). The efficacy of the bax-a MO was also tested by assessing gamma irradiation-induced apoptosis, against which it was protective (12.5 gray, Supplemental Fig. 3C) as previously described (Kratz et al., 2006). For puma MO rescue of wnt1 MO experiments, a large set of embryos was injected first with wnt1 MO, then half were re-injected with puma SB MO to ensure that the two sets received the same dose of wnt1 MO. For Bcl2-GFP injections, a plasmid containing the coding region of zebrafish Bcl2 fused to GFP was kindly provided by the authors (Smolen et al., 2007). mRNA from this construct was synthesized using the mMessage mMachine kit (Ambion, USA), purified by phenol/chloroform extraction, precipitated, and injected at 200-250 pg per embryo.

### Drug treatment

The Bcl-2 inhibitor HA14-1 (Sigma Aldrich, H8787) was dissolved in DMSO at 10 mM and further diluted in fish water immediately prior to application. The dosage of 20 μM HA14-1 was established by titration until the embryos could survive the treatment of 2.5 h without complete degeneration.

### In situ hybridization and immunohistochemistry

Embryos were grown at 28.5 °C until the desired stage, then fixed in 4% paraformaldehyde/PBS overnight. Fixed embryos were then stored in 100% methanol, or processed immediately for in situ hybridization or immunohistochemistry. In situ hybridization probes for ascl1a, ascl1b, neurog1, deltaA, and deltaB have previously been described (Amoyel et al., 2005, and references therein). Commercially available ESTs were identified by database searches for the following genes: tp53 (CK016389), p21 (CN501420), and mdm2 (EE300278)(RZPD German Resource Centre for Genome Research GmbH, Berlin, Germany). Based on published sequence information, PCR products were cloned as probe templates for the following genes: puma (Accession #DQ860151, Kratz et al., 2006), and sema3gB (Accession #AY766121, Yu and Moens, 2005). Digoxigenin-UTP labeled riboprobes were synthesized according to the manufacturer's instructions (Roche) and in situ hybridization and color development with NTB/BCIP performed as described previously (Xu et al., 1994). Embryos were then re-fixed in paraformaldehyde, cleared in 70% gycerol/PBS, and mounted for photography on a Zeiss Axiovision microscope fitted with a Zeiss Axiocam digital camera.

Primary antibodies used are anti-EphA4 (1:450, Irving et al., 1996) and anti-cleaved caspase-3 (1:250, #9661, Cell Signaling Technology). Embryos were blocked in PBS + 0.1% Tween20 and 5% goat serum. Antibodies were diluted in this blocking solution and incubated overnight at 4 °C. Detection of primary antibodies was carried out using Alexa Fluor-488, -594 or -647 goat anti-rabbit IgG (1:450, Invitrogen). The TUNEL cell death assay was performed as described previously (Berghmans et al., 2005), using the In Situ Cell Death Detection Kit, Fluorescein (Roche). Fluorescent images were captured using a Leica TCS SP2 confocal microscope.

## Results

Working on a family of novel genes, we were confounded by our inability to rescue the phenotypes caused by morpholino injection using forced expression of the wild-type protein. The phenotypes that we observed were notably similar to those described in a previous study from our laboratory, in which MO knockdown of Wnt1 led to loss of neurogenesis accompanied by spreading of hindbrain boundary marker expression (Amoyel et al., 2005). We therefore re-examined these findings in light of emerging evidence that MOs can have off-target effects due to Tp53-mediated activation of cell death pathways(Robu et al., 2007).

### Wnt1 morphant embryos have extensive, Tp53-dependent cell death

**Fig. 1:**
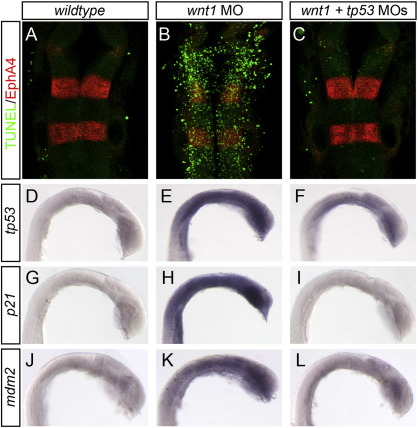
Wnt1 morpholino induces Tp53-dependent apoptosis. Uninjected (A, D, G, J), wnt1 MO-injected (B, E, H, K), and wnt1 + tp53 MO-injected (C, F, I, L) embryos were stained to detect dying cells (A–C, TUNEL, green channel), segment-specific marker EphA4 (A–C, red channel), and Tp53 transcriptional targets tp53 (D–F), p21 (G–I), and mdm2 (J–L). Wnt1 MO-injected embryos show increased cell death (compare A and B, green channel), which is rescued when tp53 MO is co-injected (compare B and C). Tp53 targets are induced in wnt1 MO-injected embryos compared to uninjected controls (compare E, H, K with D, G, J), and this expression is restored to control levels when tp53 MO is co-injected (compare E, H, K and F, I, L).

Zebrafish embryos at the 1–4 cell stage were injected with wnt1 or wnt1 + tp53morpholinos (Amoyel et al., 2005; Langheinrich et al., 2002). The embryos were grown until 22 h post-fertilization (hpf) under standard conditions, fixed, and processed for TUNEL to reveal apoptotic cells. We found that uninjected embryos have very few TUNEL-positive cells, while wnt1 morpholino-injected embryos display extensive TUNEL labeling throughout the CNS (85%, *n* = 27, Fig. 1A versus B, green channel). tp53 morpholino co-injection rescued the incidence of cell death, reducing TUNEL labeling to control levels (100%, *n* = 30). These data show that the wnt1 morpholino induces cell death in the CNS, and this appears to be acting via the Tp53 pathway.

Tp53 is a tightly regulated transcriptional regulator with downstream targets that include tp53 itself, mdm2, and the CDK inhibitor p21 (el-Deiry et al., 1993; Gartel and Tyner, 2002; Kannan et al., 2001a; Vogelstein et al., 2000). To reveal the extent of Tp53 activation in wnt1 morphant embryos, we examined the expression pattern of these Tp53 target genes. As previously described, basal levels of these markers are barely detectable in uninjected embryos (Fig. 1D, G, J, and Chen et al., 2005; Robu et al., 2007). Upon injection of wnt1 MO, transcripts for tp53 (95%, *n* = 20), mdm2 (94%, *n* = 18), and p21 (100%, *n* = 18) are all strongly induced broadly throughout the anterior region of the embryo, including the CNS (Fig. 1D–L). As expected, these Tp53 targets are reduced to near-control levels when tp53 morpholino is co-injected. These data confirm the induction of Tp53 activity in wnt1 morphants.

### Wnt1 morphant phenotypes are rescued by tp53 knockdown

**Fig. 2:**
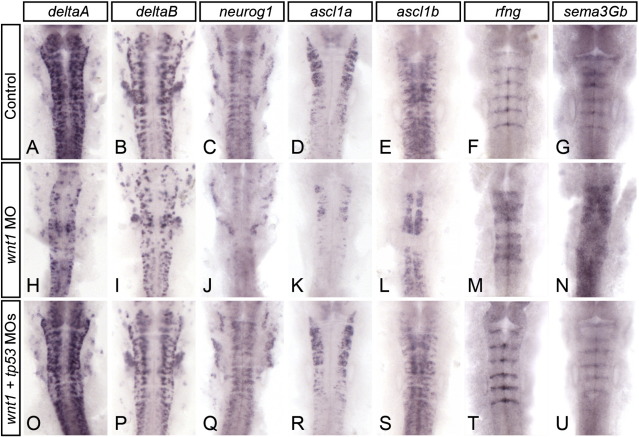
Tp53 knockdown rescues wnt1 MO phenotypes. In situ hybridization was performed to detect deltaA (A, H, O), deltaB (B, I, P), neurog1 (C, J, Q), ascl1a (D, K, R), ascl1b (E, L, S), rfng (F, M, T), and sema3gb (G, N, U) in embryos injected with control (A–G), wnt1 (H–N), and wnt1 + tp53 (O–U) morpholinos. Loss of expression of deltaA, deltaB, neurog1, ascl1a, and ascl1b in the hindbrain of wnt1 morphant embryos (compare A–E with H–L) is rescued when Tp53 is knocked down (compare H–L with O–S). Expanded expression of boundary markers rfng and sema3gb in wnt1 morphant embryos (compare F and G with M and N) is also rescued by Tp53 knockdown (compare M and N with T and U).

Expression of proneural genes neurog1 (Blader et al., 1997), ascl1a and 1b (Allende and Weinberg, 1994), and their downstream targets deltaA, B, and D (Appel and Eisen, 1998; Dornseifer et al., 1997; Haddon et al., 1998), are all reduced or lost in wnt1 morphants (Amoyel et al., 2005 and Fig. 2A–E versus H–L). The high level of cell death in the wnt1 morphants, however, prompted us to examine whether apoptosis was contributing to these phenotypes. We therefore analyzed control MO, wnt1 MO, and wnt1 + tp53 MO-injected embryos by in situ hybridization with probes for proneural genes, and deltaA and deltaB. We found that the reduction in expression of these genes in wnt1 morphants is rescued to normal levels and pattern when Tp53 levels are knocked down (Fig. 2). This result suggests that Wnt1 is dispensable for proneural gene expression in the hindbrain.

We previously found that injection of wnt1 MO led to an expansion of rhombomere boundary markers at the expense of the neurogenic non-boundary domains. Furthermore, this boundary marker expansion appeared to be dependent on the loss of proneural genes, which were thought to normally repress boundary fate in a cell-autonomous manner (Amoyel et al., 2005). Since blocking Tp53 activity in wnt1 morphants results in a restoration of proneural gene expression, we asked whether this rescue also corrected boundary cell specification, and its restriction to rhombomere interfaces. We found that MO-induced ectopic expression of the boundary markers radical fringe (rfng, Cheng et al., 2004; Qiu et al., 2004) and semaphorin-3Gb (sema3Gb, Cooke et al., 2005; Yu and Moens, 2005) (Fig. 2M,N) is completely eliminated when Tp53 levels are knocked down, and their expression becomes restricted to their normal domain (Fig. 2F,G versus T, U). These data demonstrate that Wnt1 is dispensable for the proper pattern of boundary cell fate specification.

**Fig. 3:**
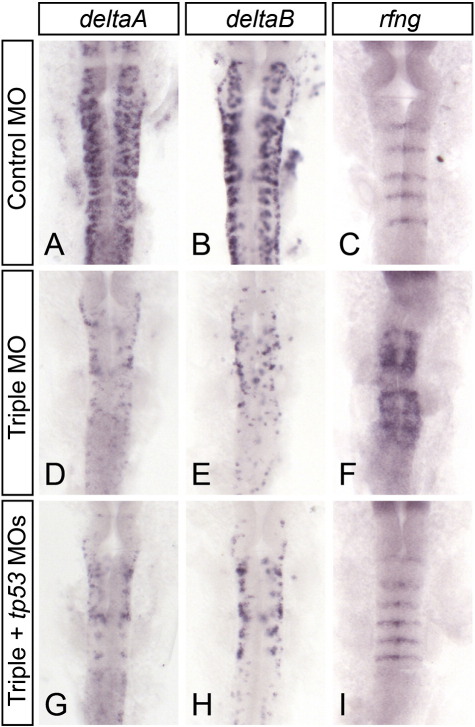
Proneural genes are not required to restrict boundary marker expression to rhombomere interfaces. Embryos injected with control (A–C), neurog1 + ascl1a + ascl1b (triple, D–F) or triple + tp53 (G–I) morpholinos were hybridized with RNA probes to deltaA (A, D, G), deltaB (B, E, H), and rfng (C, F, I). Effective proneural gene knockdown is inferred from the decrease in downstream genes deltaA and deltaB in triple MO-injected embryos (compare A and B with G and H). Boundary marker rfng expression does not expand to non-boundary regions in the absence of proneural genes when cell death is blocked (compare C and I). The expansion of rfng expression in triple proneural gene knockdown alone (compare C and F) is therefore dependent on Tp53 activity and/or apoptosis (compare F and I).

The presence of tp53 MO leads to two major changes in wnt1 knockdown embryos: cell death is blocked, and proneural gene expression is restored. Consequently, these data cannot distinguish between the original model in which proneural genes repress boundary fate (Amoyel et al., 2005) and one in which Tp53 and/or cell death is causing ectopic expression of boundary markers. To address this question directly, we performed a knockdown of all three proneural genes, while cell death was blocked with tp53 MO. If proneural genes repress boundary fate, boundary domains would expand in these proneural-depleted embryos, even when Tp53 activity is blocked. Upon injection of MOs for neurog1, ascl1a, and ascl1b and tp53 we find that proneural genes are largely knocked down, as revealed by the reduction in deltaA and deltaB expression (100%, *n* = 15 and *n* = 18, Fig. 3A,B versus G, H). Despite this, the boundary marker rfng remained restricted to rhombomere boundary domains (90%, *n* = 20, Fig. 3C versus I). These data rule out a requirement for proneural genes in the repression of boundary marker expression. As found previously (Amoyel et al., 2005), we observed that triple proneural MO injection without tp53 MO induces boundary marker expansion (100%, *n* = 18, Fig. 3C versus F).Thus, the effects of proneural gene knockdown on boundary spreading are Tp53-dependent.

**Supplemental Figure 1:**
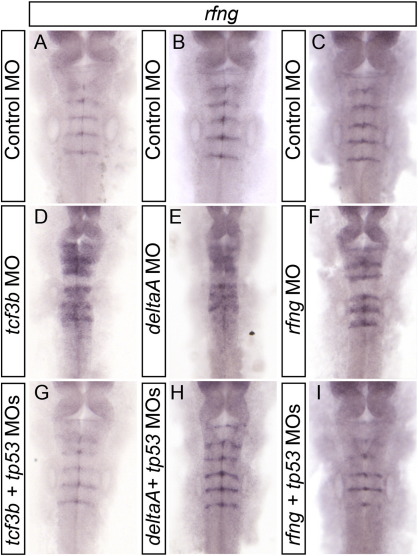
Additional morpholinos cause Tp53-dependent boundary domain expansion. Embryos injected with control (A–C), tcf3b (D), deltaA (E), rfng (F), tcf3b + tp53 (G), deltaA + tp53 (H), and rfng + tp53 (I) morpholinos, and hybridized with probe to boundary marker rfng. Tcf3b, deltaA, and rfng MOs alone induce an expansion of rfng expression (compare A–C with D–F), that is rescued when tp53 MO is co-injected (compare D–F with G–I).

We have so far shown that the phenotypes of Wnt1 and proneural gene knockdowns are dependent on Tp53 activation, rather than the specific loss of the gene products. To determine whether this was more broadly applicable, we asked whether the effect of morpholinos for other components of the putative network regulating neurogenesis and boundary restriction was also dependent on activation of Tp53. We performed a series of morpholino injections for deltaA, tcf3b, and rfng, which had previously been shown to induce boundary spreading (Amoyel et al., 2005). Morpholinos were injected alone, or in combination with tp53 MO. In all cases, boundary marker expression was expanded when the MO was injected alone, and rescued by co-injection with tp53 MO (Supplemental Fig. 1 D–F versus G–I). These data reinforce the conclusion that the boundary spreading phenotypes we have observed are due to activation of Tp53, rather than a specific effect of any particular gene knockdown.

### tp53 mutants facilitate the use of toxic morpholinos

**Supplemental Figure 2:**
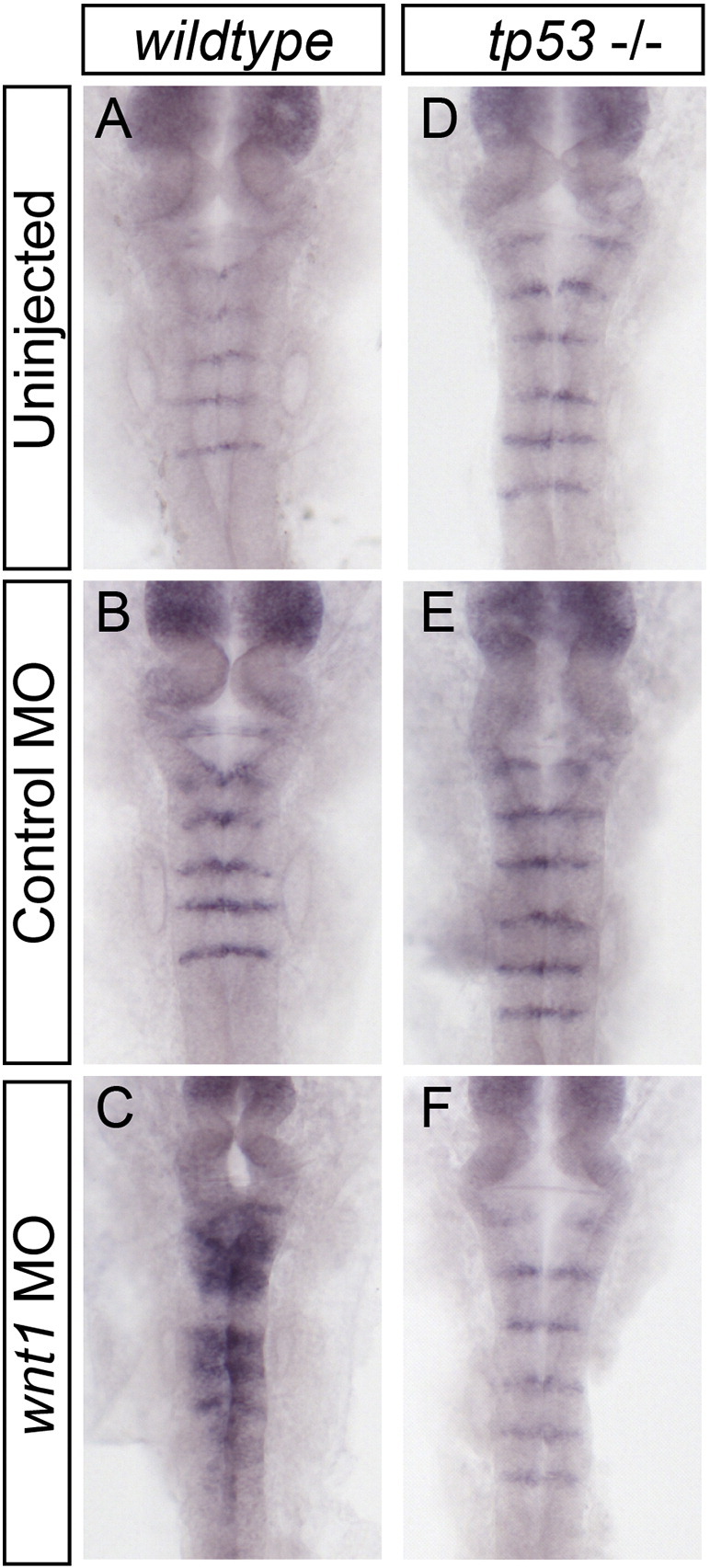
Tp53 mutant fish do not display phenotypes of morpholino toxicity. Wild-type (A, B, C) and tp53 mutant (D, E, F) embryos were injected with control or wnt1 MOs. In situ hybridization for rfng mRNA revealed extensive ectopic rfng expression in the wnt1 MO-injected wild-type embryos, while no ectopic rfng was observed in the wnt1 MO-injected tp53 mutant fish (compare C and F).

During our analysis of morpholino toxicity, we found that doses of tp53 morpholino lower than 0.79 pmol are not always effective at blocking MO toxicity, as revealed by persistent ectopic rfng expression and upregulation of p21 (data not shown). One publication (Bill et al., 2009) suggests setting the dose of tp53 MO to the same dose as the specific MO being used. However, since effective MO doses vary from gene to gene, and MOs have differing toxicity, varying the dose of tp53 MO will necessarily vary its effectiveness. To find a more reliable method to avoid tp53 activity, we used a tp53 mutant line that lacks Tp53 DNA binding activity and radiation induced apoptosis but appears to develop normally and is homozygous viable (Berghmans et al., 2005). To test whether the tp53 mutation in this line is also sufficient to abrogate morpholino toxicity, we injected clutches of tp53 homozygous embryos and wild-type embryos with the same doses of control or wnt1 MO. Whereas wnt1 morphants in the wild-type background showed the expected expansion of rfng expression at 24 hpf (100%, *n* = 19), wnt1 morphants in the tp53 mutant background showed normal expression of rfng (100%, *n* = 30), indistinguishable from control MO-injected embryos (Supplemental Fig. 2). The tp53 mutant line therefore can be used instead of tp53 morpholino co-injection, without the variability of knockdown, and permitting increased doses or multiple MOs to be used together while maintaining lower total MO levels.

### Boundary domain expansion is due to events downstream of Bcl activation

**Fig. 4:**
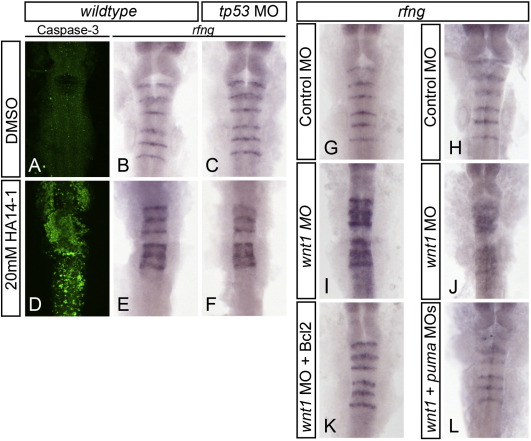
Pro-apoptotic Bcl protein activity is necessary and sufficient for ectopic expression of boundary markers. 16ss embryos treated for 2.5 h with 20 μM Bcl2 inhibitor HA14-1 undergo extensive apoptosis (anti-active caspase3 staining in green channel, compare A and D), and express ectopic rfng (compare B and E). Ectopic rfng expression induced by HA14-1 treatment is still present in HA14-1-treated tp53 morphants (compare E and F), demonstrating that Tp53 is not required. To assess the requirement for Bcl activity in MO induced ectopic rfng expression, wnt1 morphant embryos were co-injected with either Bcl2-GFP mRNA (G, I, K) or a morpholino to knockdown the pro-apoptotic Bcl gene puma (H, J, L). Both approaches rescued the expanded rfng expression (decreased ectopic rfng in K compared to I, L compared to J). All four experiments place ectopic rfng downstream of pro-apoptotic Bcl activity.

Tp53 is required for the transcriptional regulation of numerous genes independently of its function in activating the cell death pathway (Vogelstein et al., 2000). Therefore, the ectopic expression of hindbrain boundary markers in wnt1 morphants could be due to direct transcriptional regulation by Tp53, with cell death occurring as a parallel consequence of Tp53 activation. If instead this expansion of boundary marker expression in morphant embryos is due to activation of cell death pathways, then inducing cell death in the absence of Tp53 activation should give a similar phenotype. To test this, we employed a small-molecule inhibitor of anti-apoptotic Bcl proteins, HA14-1, that promotes the activation of pro-apoptotic Bcl proteins resulting in programmed cell death (Manero et al., 2006; Wang et al., 2000). Embryos were grown until 16ss, exposed to 20 μM HA14-1 or DMSO until 21ss, and then fixed. We confirmed that the drug was inducing cell death by antibody staining for active Caspase-3, an indicator of apoptosis (Nicholson et al., 1995). We found that DMSO-treated control embryos have little active Caspase-3 staining, while HA14-1-treated embryos had extensive activated Caspase-3 staining (100%, *n* = 12, compare Fig. 4A and D). This result was confirmed by TUNEL assay (data not shown). Whereas DMSO-treated embryos show a normal pattern of rfng expression, HA14-1 induces a dramatic expansion of rfng expression throughout the hindbrain except in r4 (94%, *n* = 32, Fig. 4B versus E). These data strongly suggest that activation of the cell death pathway is responsible for the expansion of boundary marker expression we see in many different morphant embryos. Importantly, HA14-1-treated embryos do not show an increase in Tp53 activity (data not shown), nor can the injection of tp53 morpholino rescue the Rfng expansion (Fig. 4E versus F), confirming that the drug is acting independently of Tp53 activity.

To confirm that the boundary domain expansion in morpholino injections was due to events downstream of Bcl protein activation, we performed the reciprocal experiment and attempted to rescue the spreading induced by the wnt1 morpholino by blocking pro-apoptotic Bcl activity. A common method for blocking Bcl-dependent apoptosis is by forced expression of the anti-apoptotic Bcl2 protein, which is known to bind to and thereby inhibit the function of pro-apoptotic Bcl proteins (Adams, 2003; Chipuk and Green, 2008). We first collected a batch of 1 cell-stage embryos, and injected half of them with mRNA encoding a Bcl2-GFP fusion (Smolen et al., 2007). We then pooled these embryos with their uninjected siblings and injected all of them with the same dose of wnt1 MO. This method ensured that wnt1 MO-injected and MO + bcl2 mRNA-injected embryos received the same dose of wnt1 MO. While all wnt1 MO alone injected embryos displayed the characteristic spreading of Rfng expression, most wnt1 MO + bcl2-GFP mRNA-injected embryos showed a rescue of this phenotype (83%, *n* = 41, Fig. 4I versus K). We also observed a rescue of the decrease in deltaA expression in the bcl2-GFP-injected embryos (data not shown). Therefore, blocking apoptosis in wnt1 morphants at the level of Bcl2 rescues the phenotypes of morpholino toxicity. These data confirm that Tp53 activity is not acting directly to induce boundary marker expression, but rather this is dependent on the cell death pathway downstream of Bcl protein activation.

### Pro-apoptotic bcl gene puma contributes to MO-induced rfng expression

Of the pro-apoptotic bcl genes, puma has been identified as a critical component in the induction of cell death and is a direct transcriptional target of Tp53 (Han et al., 2001; Jeffers et al., 2003; Nakano and Vousden, 2001; Sidi et al., 2008; Yu and Zhang, 2003; Yu et al., 2001). By in situ hybridization, puma (Kratz et al., 2006) mRNA was not detectable in 12–24 hpf wild-type embryos (data not shown). Injection of wnt1 MO, however, induced expression of puma throughout the embryo, suggesting that puma is also a Tp53 target in zebrafish (data not shown). To determine whether Puma was playing an essential role in the apoptosis caused by morpholino toxicity, we attempted to rescue the wnt1 morphant phenotype with a Puma knockdown. We injected batches of embryos with wnt1 morpholino, followed by re-injection with a splice-blocking puma morpholino (puma SB MO, Sidi et al., 2008) in half of them. While all wnt1 morphants had an expansion of the rfng domain at 24 hpf, most wnt1 MO + puma SB MO-injected embryos showed a rescue of this phenotype, especially in the posterior rhombomeres (92.5%, *n* = 40, Fig. 4J versus L). The partial nature of the rescue is likely explained by an incomplete inhibition of apoptosis, as there was residual active-caspase3 staining in the wnt1 + puma SB MO co-injected embryos (data not shown). These data indicate that Tp53 activity is acting via Puma expression to induce ectopic boundary marker expression.

### Pro-apoptotic bcl genes are required for normal boundary marker expression

**Fig. 5:**
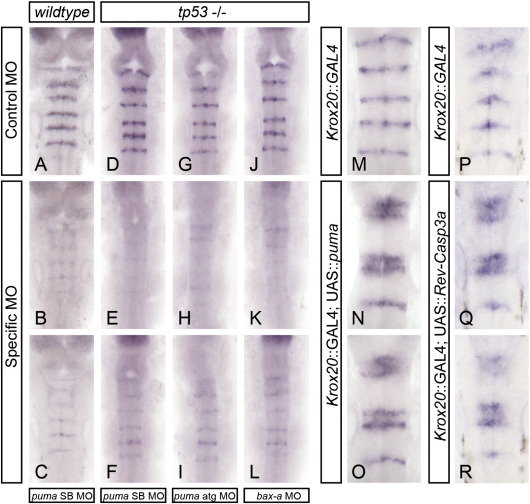
Pro-apoptotic Bcl genes puma and bax-a are required for normal boundary marker expression. Embryos injected with either of 2 different puma morpholinos (A–I) or a bax-a morpholino (JvL) show reduction or loss of rfng mRNA expression at the 21ss. Puma knockdown in wild-type (A–C) and tp53 mutant (D–I) embryos showed equivalent reductions in rfng expression (compare B, C and E, F, H, I). A krox20::GAL4 driver line was crossed to a UAS::puma line to force expression of Puma in rhombomeres 3 and 5. Double-positive embryos showed increased apoptosis (data not shown) and ectopic expression of rfng within these 2 segments (compare M and N, O). Similar ectopic rfng expression was detected when this GAL4 line was crossed to a UAS linked auto-activating Caspase3a (Rev-Casp3a, compare N, O and Q, R).

The observation that Puma is upstream of ectopic Rfng expression under MO toxicity conditions led us to ask whether there is a normal role for Puma in boundary marker expression. We found that embryos injected with puma SB + tp53 MOs showed a decrease or loss of rfng expression at rhombomere boundaries (93%, *n* = 87) compared with ctrl + tp53 MOs (0%, *n* = 56, Fig. 5A versus B, C). We repeated these injections in a tp53 mutant background to avoid any MO toxicity and additionally confirmed the specificity of this result using a second, translation blocking puma morpholino (puma ATG MO, Kratz et al., 2006): both conditions showed a reduction or loss of rfng expression at boundaries (100%, *n* = 60; 80%, *n* = 31; Fig. 5D–F and G–I). This result indicates that the pro-apoptotic Bcl gene puma plays a role in normal rhombomere boundary development. To determine if this activity affected other boundary markers, we performed in situ hybridizations for sema3Gb in Puma morphants. We found that the higher expression of sema3Gb at rhombomere boundaries is also reduced or lost when Puma is knocked down (data not shown).

We next asked whether forced expression of Puma could induce ectopic Rfng expression. We cloned the zebrafish puma coding sequence downstream of a Gal4 upstream activator sequence cassette (5xUAS). Stable transgenic UAS::puma fish were crossed to a krox20::GAL4 driver line, which expresses Gal4 in r3 and r5 (Distel et al., 2009). The resulting double transgenic embryos showed obvious cell death in r3/5 at 24 hpf, which was confirmed by anti-active-caspase-3 staining (100%, *n* = 20, data not shown), and robust puma mRNA expression in these rhombomeres (data not shown). In situ hybridization for rfng on a cohort of double transgenic embryos around 24 hpf revealed smaller r3/r5, due to cell loss, and ectopic rfng expression in non-boundary regions specifically in these rhombomeres (ectopic rfng: 94%, *n* = 16, Fig. 5M versus N,O). This experiment shows that forced expression of Puma is sufficient to induce ectopic rfng expression. Taken together, these data reveal a novel role for a pro-apoptotic bcl gene in the regulation of hindbrain boundary cells.

The puma gain- and loss-of-function phenotypes suggested two possible scenarios. The first is that they reveal a novel function for this protein, heretofore known only for its role in apoptosis. The second is that Puma is regulating rfng expression via activation of the mitochondrial apoptotic pathway. To distinguish between these possibilities, we performed experiments to either block or ectopically activate the mitochondrial pathway downstream of Puma. First, we found that a morpholino knockdown of Bax-a (Kratz et al., 2006), the direct downstream target of Puma in the apoptotic cascade, results in a reduction or loss of boundary marker expression (86%, *n* = 21, Fig. 5J versus K, L) similar to puma morphants. Second, we activated the apoptotic pathway by forced expression of an auto-activating zebrafish Caspase3a (see Materials and methods) in r3 and r5. In situ hybridization for rfng on double transgenic embryos around 24 hpf revealed ectopic rfng expression in non-boundary regions specifically in these rhombomeres (ectopic rfng: 81%, *n* = 32, Fig. 5P versus Q, R), similar to Puma forced-expression (Fig. 5N, O versus Q, R). Taken together, these data support the idea that in wild-type embryos, Puma is acting via the pro-apoptotic pathway to activate Bax-a and Caspases in regulating hindbrain boundary-specific gene expression.

## Discussion

Our findings have uncovered previously unrecognized pitfalls to the use of morpholinos for gene knockdown and suggest that these reflect a novel non-apoptotic role for pro-apoptotic genes. We re-examined the interpretation of a previous study (Amoyel et al., 2005), in light of recent revelations concerning the reliability and specificity of morpholino knockdown phenotypes. Robu et al. described a non-target related increase in Tp53-dependent apoptosis in the CNS of morpholino-injected zebrafish (Robu et al., 2007). The mechanism by which morpholinos activate Tp53 is not known but is sequence-specific (Robu et al., 2007; Wickstrom et al., 2004) and may involve cellular stress pathways. These off-target effects can mask true phenotypes and lead to erroneous conclusions regarding genetic relationships and developmental mechanisms. The majority of morpholinos that we tested can induce Tp53 dependent apoptosis, and boundary marker expansion and decreased neurogenesis (Amoyel et al., 2005) are rescued when cell death is blocked. Inducing cell death by treatment with a small-molecule inhibitor of Bcls, or forced expression of the pro-apoptotic Bcl, Puma, or Caspase3a phenocopies wnt1 morphant phenotypes, supporting the idea that activation of the cell death pathway is the major cause of the phenotypes. Reciprocally, blocking the activation of apoptosis downstream of Tp53 using Bcl2 overexpression, or a puma knockdown (Kratz et al., 2006; Sidi et al., 2008), rescues the wnt1 morpholino-induced expansion of boundary domains. These findings are consistent with the observation that a deletion mutant of wnt1 does not have decreased neurogenesis in the zebrafish hindbrain (Riley et al., 2004). We now conclude that the original interpretation of the role of Wnt1 (Amoyel et al., 2005), and by extension that of the other genes knocked down in that study, is incorrect. The results of other studies in which neurogenesis and/or boundaries are disrupted following MO-mediated knockdown need to be re-examined. Moreover, it seems unlikely that such artifacts occur only in the hindbrain.

### A novel role for pro-apoptotic genes in hindbrain boundary cells

We have shown that the pro-apoptotic bcl genes puma and bax-a are required for normal expression of markers of hindbrain boundaries. Tp53, Bcl genes, and caspases have been implicated in various non-apoptotic developmental processes in ES cell, hematopoietic, neural and muscle development (Lamkanfi et al., 2007). In many instances, the role involves a requirement for pro-apoptotic gene activity in the progression of a stem cell to a differentiation program. For example, in mouse embryonic stem cells, caspase3 activity is required to degrade the pluripotency factor NANOG in order for differentiation to occur (Fujita et al., 2008). While genes in the apoptotic cascade have been implicated individually in non-apoptotic events, there is little if any evidence for a pathway from Tp53 to Bcl to caspases in cell differentiation. Indeed, we find that Tp53 is not required for normal development of hindbrain boundary cells.

Chang et al. have suggested the balance of pro- and anti-apoptotic bcl proteins influences the choice of neuronal versus glial fate in the mouse cortex, independent of caspase activity (Chang et al., 2007). This is in contrast to other findings that caspase activity affects neuronal differentiation (Fernando et al., 2005). In our system, Caspase activity appears important, as the upregulation of boundary marker rfng upon Puma overexpression also occurs following forced expression of Caspase3a. One study linking Bcl activity to a non-apoptotic role in development (Weber and Menko, 2005) has provided a possible answer to an important question: how can a cell activate the apoptotic pathway and not die? Their observation that numerous endogenous caspase inhibitors, IAPs and survivins, are expressed concomitant with caspase activation may reveal the mechanism by which a cell modulates the strength or character of the response to pro-apoptotic proteins, resulting in signaling rather than death. Numerous cell death inhibitors are present in the zebrafish hindbrain (Thisse and Thisse, 2004) that can play such a role.

Identification of the upstream regulators and downstream targets of Puma and Caspase3 is now an important goal for understanding how hindbrain boundaries develop. The segmentally expressed receptor tyrosine kinase EphA4 is required for zebrafish hindbrain boundary formation (Cooke et al., 2005; Xu et al., 1995) and rfng expression (Gerety unpublished) and might therefore activate, or be dependent on, Puma and downstream Caspase3 activity. Several studies support the intriguing possibility that caspases modulate Eph receptor activity or vice versa (Depaepe et al., 2005; Furne et al., 2009). Additionally, Notch signaling has been implicated in the maintenance of zebrafish hindbrain boundary cells (Cheng et al., 2004; Qiu et al., 2009, and Gerety unpublished; Riley et al., 2004) and has been linked to apoptotic genes in other contexts (Kannan et al., 2001b; Okuyama et al., 2004). Interactions between the Eph/ephrin, Notch, and apoptotic pathways are therefore potentially relevant avenues for further investigations.

### Off-target effects of morpholinos are not restricted to loss of cells

Loss of a cell population, reflected by a loss of marker expression, is a common outcome of MO-induced non-specific cell death (Robu et al., 2007). Our study extends this by demonstrating that the non-specific effects of morpholinos can include increased expression of tissue-specific markers. Notably, the pattern of ectopic boundary marker expression seemed to provide evidence for a regulatory network, since it is temporally and spatially restricted, and correlates with the decreased expression of proneural genes normally expressed in a reciprocal domain. However, we find that spreading of boundary marker expression is not due to loss of proneural expression but rather is dependent upon activation of the cell death pathway, as it was rescued by Tp53 knockdown. Our findings suggest that the reason why morpholinos can non-specifically induce ectopic gene expression is that pro-apoptotic proteins have a normal role in upregulating specific genes; morpholino-induced stress activates pro-apoptotic proteins via Tp53, whereas another activating pathway normally acts at hindbrain boundaries. There is extensive evidence that a sufficient level of activation of Tp53 and downstream genes is required to trigger cell death (Vousden and Prives, 2009). It is therefore possible that changes in gene expression and cell death are not necessarily concomitant events. Consequently, in situations in which pro-apoptotic pathways regulate cell differentiation, even morpholinos for which elevated cell death is not detected could nevertheless lead to changes in gene expression due to non-specific activation of Tp53. In light of this, assaying Tp53 activity by analyzing downstream targets may be more rigorous than assaying for apoptosis itself.

### Not all death is non-specific

Taken together with the findings of Robu et al. (2007), we argue that two questions should be addressed in all experiments using morpholino-mediated knockdown (Box 1), regardless of whether the phenotype involves a loss or gain of gene expression: (1) is there increased Tp53 activity and/or cell death? This can be ascertained by detection of Tp53 target genes and the use of TUNEL staining or cleaved-caspase3 antibodies. (2) Does co-injection with tp53 morpholino rescue the phenotypes? If it does, it is possible that the phenotype is non-specific. However, while morpholinos can induce non-specific Tp53 activation and apoptotic cell death (Robu et al., 2007), this does not imply that all cell death phenotypes are non-specific. Numerous examples exist in which the normal outcome of a loss of gene function is cell death (Hettmann et al., 2000; Sugo et al., 2004). One example is the anti-apoptotic gene mdm2, which both inhibits the function of Tp53 and targets it for degradation (Momand et al., 1992). It is not surprising that the mouse knock-out has specific, extensive cell death (Jones et al., 1995; Montes de Oca Luna et al., 1995). More widely, trophic factors, mitogens, and their receptors are often required for cell survival, and cell death is a common outcome of abnormal cell specification. Given what we now know about the toxicity of morpholinos, their use to study genes displaying apoptotic cell death as a loss-of-function phenotype is highly problematic. A number of studies in zebrafish claim survival roles for disease candidate genes based on morphant embryo apoptosis data, and it is important that these studies be re-examined.

### Injection controls

It is clear now that the “standard control” morpholino (Gene Tools) is inadequate to establish a baseline for non-specific MO effects, because it has inherently low levels of toxicity (Eisen and Smith, 2008; Robu et al., 2007). Nevertheless, despite having only weak Tp53 activating toxicity, this MO should be used as a control for any additional non-specific morpholino effects. An alternative and commonly used control is to use mutated morpholinos, such as “5-mismatch morpholinos”. However, advances in our understanding of morpholino off-target effects now argue against this approach: off-target effects are sequence-dependent, since changing 4 bases in a morpholino can alter its toxicity (Wickstrom et al., 2004). Thus, a 5-mismatch control is unlikely to retain the same level of non-specific toxicity of its unmutated parent MO and cannot be used as a reference point for specificity. Reproducing a phenotype using a second, non-overlapping morpholino (in the context of a Tp53 co-knockdown) provides strong evidence for specificity.

### Rescue to the rescue

An important strategy for establishing the specificity of knockdown is to rescue the deficiency by providing exogenous gene product back into the system. Many studies take this approach, injecting full-length mRNAs into morphant embryos to demonstrate that the observed phenotypes are due to the specific gene knockdown. However, there are situations in which this approach is problematic, for example, where ectopic or elevated expression of a gene has a strong gain-of-function phenotype, and/or injection of mRNA will alter development prior to the process that is being studied. More sophisticated techniques such as heat shock or GAL4/UAS based overexpression (Esengil and Chen, 2008; Halpern et al., 2008) can provide a means of circumventing these difficulties and, when feasible, should be used. For GAL4/UAS approaches, this will depend upon the availability of regulatory sequences to drive the appropriate temporal and spatial expression.

With regard to the issue of toxic morpholinos, there is at least one potential problem with the overexpression rescue approach. Many genes, when overexpressed, can have survival or anti-apoptotic effects on cells. Therefore, if a morpholino loss-of-function results in cell death that is rescued by mRNA injection, can we conclude that this gene is required for cell survival? Given the prevalence of non-specific cell death, we would argue that this result is difficult to interpret as it is possible that the MO is toxic and the target has anti-apoptotic activity. This applies not only to the question of cell survival function but also to any rescued phenotype, as it may in fact be due to cell death or Tp53 activity. For example, in our previous study (Amoyel et al., 2005), forced expression of Wnt1 reversed the decrease in neurogenesis caused by morpholino knockdown of Wnt1, but this apparent rescue was likely due to the action of Wnt1 as a survival factor, which presumably counteracted the effect of Tp53 activation. It is important to emphasize that proper controls that include Tp53 co-knockdown would alert attention to the possibility of MO toxicity.

### Further controls?

The definitive control for a morpholino knockdown is a mutant line. While this positive control may appear to obviate the need for using a morpholino in the first place, the ability to generate hypomorphs, multigene knockdown embryos, and carry out first tier screening continue to make morpholinos an attractive tool. Additionally, it is not realistic at present to obtain a mutant fish line for every morpholino experiment: the efforts to generate TILLING mutants (Wienholds et al., 2003) lag well behind the generation of morphant phenotypes in need of validation. This limitation should be alleviated by recent increases in TILLING throughput, advances in targeted mutations using zinc finger nucleases, and the promise of homologous recombination (Doyon et al., 2008; Ekker, 2008; Fan et al., 2004; Meng et al., 2008). In the absence of these, approaches such as the forced expression of dominant-negative versions of the gene should be undertaken when possible to confirm specificity.

### How useful are morpholinos?

The appeal of morpholinos has been their ease of use and apparent reliability. However, the prevalence of non-specific effects poses a conundrum, particularly for the analysis of genes with unknown function: are morpholinos a sufficiently reliable tool in this pursuit? In agreement with recent reviews of the application of morpholino knockdown (Bill et al., 2009; Eisen and Smith, 2008), we would argue that they are, if carried out with the appropriate controls. Although a high proportion of morpholinos can cause non-specific apoptosis, for many it is possible to find a dose that gives a specific phenotype without such toxicity. In particular, the phenotype is likely to be specific if it still occurs following co-injection with tp53 MO, as this should block non-specific activation of the apoptotic pathway. However, as discussed above, the interpretation can be confounded if cell death is a normal consequence of loss of the gene function, or if Tp53 has a role in the process being studied. Finally, although the current study has focused on a set of non-specific effects of morpholinos in zebrafish, it is possible that analogous difficulties arise in other developmental systems and indeed do occur with other reagents that can have off-target effects leading to activation of stress pathways and Tp53 (siRNA, dsRNA, gripNAs: Kittler et al., 2007; Kulkarni et al., 2006; Robu et al., 2007; Urtishak et al., 2003).

The following are the supplementary materials related to this article

**Supplemental Figure 3:**
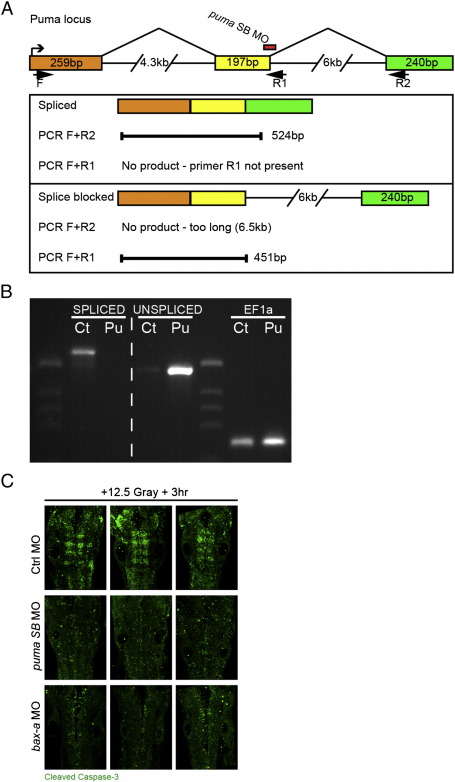
Puma and bax-a morpholino efficacy. Schematic of puma genomic locus, splicing pattern and observed spliced and unspliced products (A). RT-PCR (B) using primers to detect spliced (primers F + R2, SPLICED) and unspliced (primers F + R1, UNSPLICED) puma transcripts was performed on control MO (Ct) and puma SB MO (Pu)-injected embryos (0.56 pmol each). Loss of spliced puma (SPLICED, compare Ct and Pu) and increase in unspliced puma (UNSPLICED, compare Ct and Pu) upon puma SB MO injection was observed. PCR using primers to EF1 confirmed near-equivalent amounts of cDNA were used. Puma splice-blocking and bax-a morpholinos protect against gamma irradiation induced apoptosis (C). Control MO-injected embryos (C, Ctrl MO) show extensive Caspase3 activation (anti-active Caspase3, green) after exposure to 12.5 Gray irradiation, while both pumaSB and bax-a MO-injected embryos show much reduced levels. Embryos were irradiated at 18ss, and subsequently grown for 3 h at 28.5 °C.

Supplemental Table 1 Summary of results of all experiments. Percentages represent the indicated states (e.g., expression increased, expression normal). *n*-values represent total number of embryos analyzed. Exp values represent the number of independent experiments quantified for this table. All results were found to be statistically significant (two-tailed Fisher's Exact Test, *p* < 0.001)

## Box 1 Recommendations for performing morpholino experiments.

1. All MOs should be tested for apoptotic phenotypes by TUNEL, cleaved Caspase3 staining, and in situ hybridization for tp53 targets.
2. All MO experiments should be done in parallel with *tp53* MO co-injected, or *tp53* mutant samples to catch cell-death related phenotypes and “non-typical” cell-death associated phenotypes.
3. Any phenotype that is rescued by Tp53 knockdown cannot reliably be studied by morpholino knockdown—*seek alternative approaches*.
4. Standard control morpholino, and uninjected controls, should be used in every injection experiment.
5. A second, non-overlapping MO should be used to replicate the phenotype.
6. Whenever feasible, supporting evidence should be provided, either by dominant negatives, forced-expression rescue, or gain of function phenotypes (with caveats described in text).
