# Supplementary material for: Morpholino artifacts provide pitfalls and reveal a novel role for pro-apoptotic genes in hindbrain boundary development
Source: Dev Biol. 2011 Feb 15;350(2):279–89. doi: 10.1016/j.ydbio.2010.11.030 (PMC3111810; doi:10.1016/j.ydbio.2010.11.030)
Supplement: Supplemental Table 1 — Summary of results of all experiments. Percentages represent the indicated states (e.g., expression increased, expression normal). n-values represent total number of embryos analyzed. Exp values represent the number of independent experiments quantified for this table. All results were found to be statistically significant (two-tailed Fisher's Exact Test, p < 0.001) [file mmc1.doc]

| **Figure 1** |  |  |  |  |
| --- | --- | --- | --- | --- |
|  | **TUNEL staining increased** | ***tp53* expression increased** | ***mdm2* expression increased** | ***p21* expression increased** |
| **wildtype** | 0% (n=20, 2 exps) | 0% (n=21, 2 exps) | 0% (n=25, 2 exps) | 0% (n=20, 2 exps) |
| ***wnt1* MO** | 85% (n=27, 2 exps) | 95% (n=20, 2 exps) | 94% (n=18, 2 exps) | 100% (n=18, 2 exps) |
| ***wnt1+tp53* MOs** | 0% (n=30, 2 exps) | 0% (n=35, 2 exps) | 0% (n=28, 2 exps) | 0% (n=25, 2 exps) |
|  |  |  |  |  |
| **Figure 2** |  |  |  |  |
|  | ***deltaA* expression normal** | ***deltaB* expression normal** | ***neurog1* expression normal** | ***ascl1a* expression normal** |
| **control MO** | 96% (n=46, 2 exps) | 100% (n=51, 2 exps) | 96% (n=45, 2 exps) | 100% (n=36, 2 exps) |
| ***wnt1 MO*** | 0% (n=43, 2 exps) | 0% (n=40, 2 exps) | 0% (n=42, 2 exps) | 0% (n=36, 2 exps) |
| ***wnt1+ tp53* MOs** | 89% (n=46, 2 exps) | 100% (n=48, 2 exps) | 89% (n=53, 2 exps) | 100% (n=42, 2 exps) |
|  |  |  |  |  |
|  | ***ascl1b* expression normal** | ***rfng* expression normal** | ***sema3Gb* expression normal** |  |
| **control MO** | 100% (n=48, 2 exps) | 100% (n=72, 3 exps) | 100% (n=25, 2 exps) |  |
| ***wnt1 MO*** | 0% (n=50, 2 exps) | 1% (n=91, 3 exps) | 0% (n=36, 2 exps) |  |
| ***wnt1+ tp53* MOs** | 100% (n=55, 2 exps) | 91% (n=82, 3 exps) | 97% (n=36, 2 exps) |  |
|  |  |  |  |  |
| **Figure 3** |  |  |  |  |
|  | ***deltaA* expression normal** | ***deltaB* expression normal** | ***rfng* expression normal** |  |
| **control MO** | 100% (n=20, 2 exps) | 100% (n=18, 2 exps) | 100% (n=19, 2 exps) |  |
| ***neurog1/ascl1a/ascl1b MOs (*triple*)*** | 0% (n=16, 2 exps) | 0% (n=15, 2 exps) | 0% (n=18, 2 exps) |  |
| ***neurog1/ascl1a/ascl1b* MOs *(*triple*) + tp53* MO** | 0% (n=15, 2 exps) | 0% (n=18, 2 exps) | 90% (n=20, 2 exps) |  |
|  |  |  |  |  |
| **Figure 4** |  |  |  |  |
|  | **active Caspase3 high** | ***rfng* expression expanded** |  |  |
| **DMSO** | 0% (n=14, 2 exps) | 0% (n=31, 2 exps) |  |  |
| **HA14-1** | 100% (n=12, 2 exps) | 94% (n=32, 2 exps) |  |  |
| **DMSO + tp53 MO** | nd | 0% (n=12, 2 exps) |  |  |
| **HA14-1 + tp53 MO** | nd | 92% (n=12, 2 exps) |  |  |
|  |  |  |  |  |
|  | ***rfng* expression normal** |  |  |  |
| **control MO** | 100% (n=45, 2exps) |  |  |  |
| ***wnt1 MO*** | 0% (n=50, 2 exps) |  |  |  |
| ***wnt1 MO + bcl2* mRNA** | 83% (n=41, 2 exps) |  |  |  |
|  |  |  |  |  |
|  | ***rfng* expression normal in posterior rhombomeres** |  |  |  |
| **control MO** | 100% (n=45, 2exps) |  |  |  |
| ***wnt1 MO*** | 0% (n=50, 2 exps) |  |  |  |
| ***wnt1 MO + puma* SB MO** | 93% (n=40, 2 exps) |  |  |  |
|  |  |  |  |  |
| **Figure 5** |  |  |  |  |
| **in wildtype embryos** | ***rfng* expression weak/lost** |  |  |  |
| **Control + tp53 MOs** | 0% (n=56, 3 exps) |  |  |  |
| ***puma* SB *+ tp53* MOs** | 93% (n=87, 3 exps) |  |  |  |
|  |  |  |  |  |
| **in tp53 -/- embryos** | ***rfng* expression weak/lost** |  |  |  |
| **control MO** | 0% (n=52, 2 exps) |  |  |  |
| ***puma SB* MO** | 100% (n=60, 2 exps) |  |  |  |
| ***puma ATG* MO** | 80% (n=31, 2 exps) |  |  |  |
|  |  |  |  |  |
|  | ***rfng* expression weak/lost** |  |  |  |
| **control MO** | 0% (n=30, 2 exps) |  |  |  |
| ***bax-a MO*** | 86% (n=21, 2 exps) |  |  |  |
|  |  |  |  |  |
|  | ***rfng* expression ectopic** |  |  |  |
| ***krox20::*GAL4** | 0% (n=23, 2 exps) |  |  |  |
| ***krox20::*GAL4*;*UAS*::puma*** | 94% (n=16, 2 exps) |  |  |  |
|  |  |  |  |  |
|  | ***rfng* expression ectopic** |  |  |  |
| ***krox20::*GAL4** | 0% (n=30, 2 exps) |  |  |  |
| ***krox20::*GAL4*;*UAS*::Rev-Casp3a*** | 81% (n=32, 2 exps) |  |  |  |
|  |  |  |  |  |
| **Supplemental figure 1** |  |  |  |  |
|  | **rfng expression normal** |  |  |  |
| **control MO** | 100% (n=18, 2 exps) |  |  |  |
| ***tcf3b* MO** | 0% (n=26, 2 exps) |  |  |  |
| ***tcf3b + tp53 MOs*** | 85% (n=20, 2 exps) |  |  |  |
|  |  |  |  |  |
|  | ***rfng* expression normal** |  |  |  |
| **control MO** | 100% (n=29, 2 exps) |  |  |  |
| ***deltaA MO*** | 0% (n=20, 2 exps) |  |  |  |
| ***deltaA + tp53* MOs** | 100% (n=25, 2 exps) |  |  |  |
|  |  |  |  |  |
|  | ***rfng* expression normal (not ectopic)** |  |  |  |
| **control MO** | 100% (n=37, 2 exps) |  |  |  |
| ***rfng MO*** | 17% (n=24, 2 exps) |  |  |  |
| ***rfng + tp53* MOs** | 92.5% (n=27, 2 exps) |  |  |  |
|  |  |  |  |  |
| **Supplemental figure 2** |  |  |  |  |
|  | ***rfng* expression normal** | ***rfng* expression normal** |  |  |
|  | **wildtype embryo** | ***tp53 -/- embryo*** |  |  |
| **uninjected** | 100% (n=30, 2 exps) | 100% (n=16, 2 exps) |  |  |
| **control MO** | 100% (n=25, 2 exps) | 100% (n=34, 2 exps) |  |  |
| ***wnt1 MO*** | 0% (n=19, 2 exps) | 100% (n=30, 2 exps) |  |  |
|  |  |  |  |  |
| **Supplemental figure 3** |  |  |  |  |
|  | **active caspase high** |  |  |  |
| **control MO + 12.5 gray** | 100% (n=13) |  |  |  |
| ***puma* SB MO + 12.5 gray** | 0% (n=10) |  |  |  |
| ***bax-a* MO + 12.5 gray** | 0% (n=10) |  |  |  |
|  |  |  |  |  |
